# Identification of Damping of Spruce Wood (*Picea abies*) under Various Levels of Moisture Content Using Time-Scale Decomposition

**DOI:** 10.3390/polym16101313

**Published:** 2024-05-08

**Authors:** Miran Merhar

**Affiliations:** Department of Wood Science and Technology, Biotechnical Faculty, University of Ljubljana, Jamnikarjeva 101, 1000 Ljubljana, Slovenia; miran.merhar@bf.uni-lj.si; Tel.: +386-1-320-36-29

**Keywords:** damping factor, wavelet transform, logarithmic decrement, relative humidity, impulse response function, time response, natural frequency, frequency response function, Morlet wavelet, vibration

## Abstract

The damping of spruce wood is analysed at different moisture content levels for the first three vibration modes of tangentially and radially vibrating samples. Two methods were used to determine the damping. The first was the vibration envelope fitting as an improved version of the well-known logarithmic decrement, and the second was the newer and recently increasingly used wavelet transform. Both methods showed that the damping of spruce wood first decreases and then increases with moisture content, with the damping in the first vibration mode being about 9% higher in the radial direction than in the tangential direction. In the second and third vibration modes, the damping in the tangential direction was higher than in the radial direction by about 10% and 8.8%, respectively. The measured damping factors from the envelope fitting had, on average, 15.9% higher values than those from the wavelet transform. It can be concluded from the results that the wavelet transform is more accurate for determining the damping factor, as it enables the decoupling of multi-degree of freedom systems if mode coupling is present.

## 1. Introduction

Every lightly damped structure begins to vibrate when it is exposed, either permanently or temporarily, to a certain time-varying excitation [1]. While vibrations can be desirable or undesirable, the time course of the vibration amplitude is influenced by many factors, including damping, which can be modelled using different models depending on the damping mechanism, whereby damping is divided into hysteretic and viscous damping. Hysteretic damping includes damping due to the microstructure of the material, while viscous damping depends on the vibration velocity. Thus, in the real world, the phenomenon of damping is an aggregate of various causes of damping, which can be evaluated as structural damping [1,2], where the damping is represented by a damping factor, evaluated as the proportion of critical damping.

Wood damping has been the subject of numerous studies investigating the damping of different types of wood under different conditions. As wood is used in a variety of industries, from construction to musical instruments, knowledge of the mechanical properties of wood and its damping characteristics is essential. The mechanical properties of CLT panels, as well as the damping properties, were investigated by Lu et al. [3]. The effects of moisture content on mechanical and damping properties were studied by Bremaud [4,5], Bremaud and Gril [6], and Ahmed et al. [7]. When the wood is thermally modified, the damping is generally reduced, as confirmed by Zauer et al. [8], who investigated the effects of thermal modification on the damping of beech wood. Mania and Skrodzka [9] and Danihelova et al. [10] investigated the effect of thermal modification on the damping of spruce wood, Stanciu et al. [11] investigated the effect of ammonia treatment; and Buchelt et al. [12] investigated the influence of thermal modification on damping and on the modulus of elasticity at different moisture contents. The damping properties of different tree species used for musical instruments were studied by Wegst [13], Merhar and Humar [14], Sproßmann et al. [15], and Gurau et al. [16]. Temperature also plays an important role in damping the vibrations of wood. The influence of heating on the damping properties of maple and Douglas fir was studied by Mihalcica et al. [17] and Jiang et al. [18], who found that damping first increases and then decreases with increasing temperature.

Damping can be determined in different ways, either in the time domain or in the frequency domain [2]. Among the methods in the time domain for systems with a single degree of freedom (SDOF), the logarithmic decrement method is the simplest, while for systems with multiple degrees of freedom (MDOF), the Smith least squares algorithm (SLS) [19] and the least complex exponential squares method (LSCE) are popular. Among the methods in frequency space, the 3 dB method is the simplest. It can be applied to systems with one or more degrees of freedom in lightly coupled modes with minimal crossover. However, for oscillating modes where the frequencies are very close to each other or for strongly damped systems, the 3 dB method is rather inaccurate, as the frequency response function (FRF) of the individual modes influences the FRF values of the other modes. More accurate results can be achieved using curve fitting methods.

If an MDOF system can be considered a superposition of several SDOF systems that are more or less localised, the signal can be decomposed using specific functions to obtain a time-scale representation instead of a time-frequency representation. When the time shift is replaced by a translation and the frequency by dilation or scaling, the decomposition on the time scale results in a wavelet transform (WT). The first researchers in this field were Morlet [20], who used wavelet transform in seismology. Since then, the method has developed rapidly [21] and is used for a variety of purposes. Today, the wavelet transform is used to determine various modal parameters. For example, the method can be used to determine the damping [19,22,23,24], the natural frequencies of oscillations [25,26,27], or the mode shapes [28]. The WT has thus proven to be a very useful and accurate method for determining various modal parameters, whereby it can be used to filter out the influence of environmental disturbances effectively or as a filter for extracting a particular vibration mode [19,23,29].

As mentioned, much research has been conducted on the damping of wood. What all these studies have in common are the methods used. Almost all authors have used either the logarithmic decrement or the 3 dB method to determine damping, both of which have a common characteristic: they are inaccurate for coupled systems.

This article aims to present the determination of the damping of wood on the first three bending vibration modes using the wavelet transform, which has received little attention to date despite the extensive literature on WT. In contrast, many papers in the literature research the damping of wood under various conditions [3,4,5,6,9,12,14,15,17], using the predominant inaccurate methods being based on either the logarithmic decrement or the 3 dB method. The aim is also to compare the wavelet transform with the method of vibration envelope fitting as an improved version of the well-known logarithmic decrement for damping determination in the first vibration mode. In addition, the degree of inaccuracy of the second method is determined, and the usefulness of the wavelet transform for determining the damping of wood under different conditions is confirmed.

## 2. Materials and Methods

### 2.1. Specimen Preparation

**Figure 1:**
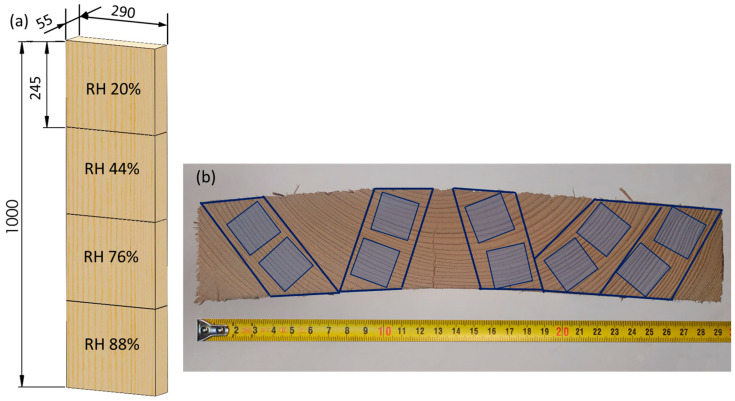
Schematic of cutting plan: (**a**) cutting from board; (**b**) specimen distribution.

A 1 m long spruce board (*Picea abies*) with a cross-section of 290 mm × 55 mm and constant growth ring width was cut into 4 smaller pieces of 245 mm length, from which 10 strictly oriented samples of 245 mm × 22 mm × 22 mm were cut (Figure 1). The individual groups of 10 samples were then equilibrated to a constant sample mass at 22 °C and relative humidity (RH) of 20%, 44%, 76%, and 88%. After the final equilibration of the samples, they were cut to a final size of 200 mm × 20 mm × 20 mm. Additional samples were used to determine the equilibrium moisture content (EMC) of the wood for each combination of relative humidity. They were weighed after equilibration, then dried to absolute dryness at 103 °C and weighed again to determine the moisture content. The equilibrium moisture content of the samples at relative humidities of 20%, 44%, 76%, and 88% was 7.2%, 9.9%, 13.3%, and 19.1%, respectively.

### 2.2. Experimental Procedures

#### 2.2.1. Vibration Measurements

The samples were freely supported at a distance of 0.22 *L* and 0.77 *L* (*L*-length) and excited with a hammer to vibrate freely at their natural frequencies. The aim was to excite the first three bending vibration modes. Each sample was first excited in the radial direction and then in the tangential direction. The sample was excited 10 times, and the time responses were measured using a Bruel & Kjaer type 4939 microphone, an NI-USB-6361 DAQ card, and LabVIEW software with a sampling rate of 50 kHz and an acquisition time of 1 s. The time recordings were then analysed using the LabVIEW software. An average frequency spectrum with a resolution of 1 Hz was then generated from 10 repeated time measurements using a fast Fourier transformation (FFT), and the frequencies for the first three vibration modes were determined.

#### 2.2.2. Damping Determination Based on Fitting the Oscillation Envelope

The first method to determine the damping factor for the first vibration mode was logarithmic decrement or fitting the oscillation envelope. When using logarithmic decrement, the damping factor is determined from two or more time-positioned amplitudes of oscillations in the system with one degree of freedom, defined as [2]:

(1) $$\delta_{n}=ln\frac{X_{i}}{X_{i+n}}=\frac{2\;n\;\pi \;\zeta }{\sqrt{1-\zeta^{2}}}.$$

**Figure 2:**
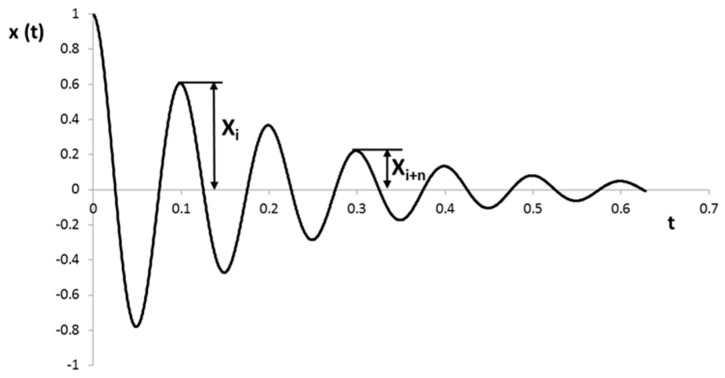
Time response.

The term *δ_n_* is called the logarithmic decrement, *ζ* is the damping factor, and *X_i_* and *X_i+n_* are the amplitudes at locations *i* and *i + n*, respectively, as shown in Figure 2.

This method is reliable and accurate in a linear system with one degree of freedom. However, if the time course of the oscillation is influenced by various factors, such as the coupling of other vibration modes or measurement noise, the time response does not take on the ideal theoretical form, which means that the method is no longer sufficiently accurate. The method can be improved by taking several consecutive vibration amplitudes and then fitting the envelope using the least squares method, which was done in this experiment.

The measured time response of the damped natural vibration was considered an impulse response function with one degree of freedom, which can be written as follows:

(2) $$x\left(t\right)=A_{0}e^{-\zeta \omega t}\cos\left(\sqrt{1-\zeta^{2}}\omega t-\phi \right),$$

where *A*_0_ is the residual amplitude, *ζ* is the damping factor, *ω* is the natural frequency of the undamped vibration, and *ϕ* is the phase shift. For fitting the envelope, the first part of the equation is considered:

(3) $$A\left(t\right)=A_{0}e^{-\zeta \omega t},$$

where the damping factor is determined from an exponential fit using the least squares method.

#### 2.2.3. Damping Determination Based on Wavelet Transform

The second method for determining the damping factor was a wavelet transform, which was explained in detail by Staszewski [19]. The wavelet transform, which provides a time-frequency representation of the time signal *x*(*t*) by a linear transformation, can be written as [19,21]:

(4) $$\left(W_{g}x\right)\left(a,b\right)=\frac{1}{\sqrt{a}}\;\int_{-\infty }^{+\infty }x\left(t\right)g^{*}\left(\frac{t-b}{a}\right)dt,$$

where *a* is the scale/dilation parameter that determines the width of the wavelet, and *b* is the translation parameter defining the position of the wavelet function in the time domain, while g*(*t*) is the conjugate complex function of the basic wavelet function *g*(*t*). Each wavelet transform is normalised by the factor $1/\sqrt{a}$, which ensures that the integral energy is independent of the dilation parameter *a*.

The wavelet function *g*(*t*) must satisfy the admissibility condition [21]:

(5) $$C_{g}=\int_{-\infty }^{+\infty }\frac{{\left|G\left(\omega \right)\right|}^{2}}{\left|\omega \right|}d\omega <\infty ,$$

where *G*(*ω*) is the Fourier transform of *g*(*t*). To satisfy the time-frequency localisation of *g*(*t*), the wavelet must also satisfy the following condition:

(6) $$\int_{-\infty }^{+\infty }\left|g\left(t\right)\right|dt<\infty .$$

Similarly, the function *x*(*t*) analysed must decay to zero over time to satisfy the condition:

(7) $$\int_{-\infty }^{+\infty }{\left|x\left(t\right)\right|}^{2}dt<\infty .$$

If the above conditions are satisfied, the signal *x*(*t*) can be regenerated by an inverse wavelet transform as:

(8) $$x\left(t\right)=\frac{1}{C_{g}}\int_{-\infty }^{+\infty }\int_{-\infty }^{+\infty }\left(W_{g}x\right)\left(a,b\right)\frac{1}{\sqrt{a}}g^{*}\left(\frac{t-b}{a}\right)\frac{da\;db}{a^{2}}.$$

If the signal *x*(*t*) can be written as a superposition of signals, the wavelet transform can also be written as a linear superposition:

(9) $$\left(W_{g}\overset{N}{\underset{i=1}{\sum }}\alpha_{i}x_{i}\right)\left(a,b\right)=\overset{N}{\underset{i=1}{\sum }}\alpha_{i}(W_{g}x_{i})\left(a,b\right).$$

In the case of the function *x*(*t*), which describes the vibration with the frequency *ω*, the following relationship applies:

(10) $$a=\frac{\omega_{0}}{\omega },$$

where *ω*_0_ is the wavelet frequency.

An alternative notation of wavelet transform can be obtained by transforming the signal *x*(*t*) and the wavelet function *g*(*t*) in frequency space:

(11) $$\left(W_{g}x\right)\left(a,b\right)=\sqrt{a}\;\int_{-\infty }^{+\infty }X\left(\omega \right)G^{*}\left(a\omega \right)e^{j\omega b}d\omega ,$$

where *X*(*ω*) and G*(*aω*) are the Fourier transforms of *x*(*t*) and g*(*t*), respectively.

Depending on the purpose of the wavelet transform, various wavelet functions can be used [29,30,31], while the Morlet wavelet has proven to be very useful for determining the modal parameters of vibrating systems:

(12) $$g\left(t\right)=e^{j\omega_{0}t}e^{-t^{2}/2}.$$

Here, *ω*_0_ is the wavelet frequency, while the spectrum of a dilated Morlet wavelet in frequency space is defined as:

(13) $$G\left(a\omega \right)=e^{-{\left(a\omega -\omega_{0}\right)}^{2}}.$$

However, since the Morlet wavelet does not fulfil the admissibility conditions (Equation (5)) over the entire frequency range, as *G*(0) > 0 results in *Cg* = ∞, values *ω*_0_ > 5 are used in practice.

In certain cases of a distributed mass system, the system can be treated as a decoupled multi-degree of freedom system, where the time response of the free vibration can be written as the sum of the individual single-degree of freedom system responses:

(14) $$x\left(t\right)=\overset{N}{\underset{i=1}{\sum }}A_{i}e^{-\zeta_{i}\omega_{i}t}\cos\left(\sqrt{1-{\zeta_{i}}^{2}}\omega_{i}t-\phi_{i}\right),$$

where *Aj* is residue amplitude, *ζ_j_* is damping ratio, *ω_j_* is the undamped angular frequency, and *ϕ_j_* is the phase shift for each decoupled mode *i*. Considering that the response can also be written as the sum of analytical signals, where only the real part is taken, Equation (14) can also be written as:

(15) $$x\left(t\right)=\overset{N}{\underset{i=1}{\sum }}A_{i}e^{-\zeta_{i}\omega_{i}t}e^{\left(\sqrt{1-{\zeta_{i}}^{2}}\omega_{i}t-\phi_{i}\right)j}.$$

If only the modulus of the wavelet transform of the function *x*(*t*) (Equation (15)) is required, the solution can be approximated as:

(16) $$\left|\left(W_{g}\overset{N}{\underset{i=1}{\sum }}x_{i}\right)\left(a,b\right)\right|\approx \overset{N}{\underset{i=1}{\sum }}A_{i}e^{-\zeta_{i}\omega_{i}b}\;\left|G{\;}^{*}\left(\pm j\;a_{i\;}\omega_{i}\sqrt{1-{\zeta_{i}}^{2}}\right)\right|.$$

Further, taking only a specific vibration mode *i* with a natural frequency *ω_i_*, each vibration mode can be decoupled into a single mode:

(17) $$\left|\left(W_{g}x_{i}\right)\left(a_{i},b\right)\right|\approx A_{i}e^{-\zeta_{i}\omega_{i}b}\;\left|G{\;}^{*}\left(\pm j\;a_{i\;}\omega_{i}\sqrt{1-{\zeta_{i}}^{2}}\right)\right|.$$

Applying the logarithm to Equation (17), the final solution is:

(18) $$\ln\left|\left(W_{g}x_{i}\right)\left(a,b\right)\right|\approx -\zeta_{i}\omega_{i}b+\ln\left(A_{i}\left|G{\;}^{*}\left(\pm j\;a_{i\;}\omega_{i}\sqrt{1-{\zeta_{i}}^{2}}\right)\right|\right).$$

Equation (18) can be used to determine the damping factor *ζ_i_* for each decoupled mode from the slope of the straight line of the wavelet modulus cross-section $\left|\left(W_{g}x_{i}\right)\left(a_{i},b\right)\right|$, for a given value of the dilation parameter *a_i_*, related to the natural frequency *ω_i_* of the system, plotted on a semi-logarithmic scale.

The curve of the maximum value of the WT modulus in the time-frequency plane is called the wavelet ridge *a_r_*(*b*), and the modulus values corresponding to the points in the curve are called the wavelet skeleton. There are different algorithms for ridge extraction. The most common one uses the local maxima of the WT modulus, which provides accurate values only for linear ridges. More precise values can be obtained from the phase function, as proposed by Tchamitchian and Toressani [32],

(19) $$\frac{\partial \Omega \left(a,b\right)}{\partial a}=0,$$

where Ω(*a*,*b*) is the phase of the wavelet transform.

In the experiment, the natural frequency of the damped oscillation *ω* was first determined from the frequency spectrum, then the corresponding dilation factor *a* was calculated, and finally, the damping factor was evaluated using Equation (18). The entire analysis was conducted using LabView software, in which the corresponding analysis code was programmed.

### 2.3. Statistical Analysis

The significant differences in damping factors were verified by the ANOVA (F-test) analysis using SPSS software, where 5% was taken for the level of significance (*p*-value).

## 3. Results and Discussion

### 3.1. Damping Factor in 1st Vibration Mode

**Figure 3:**
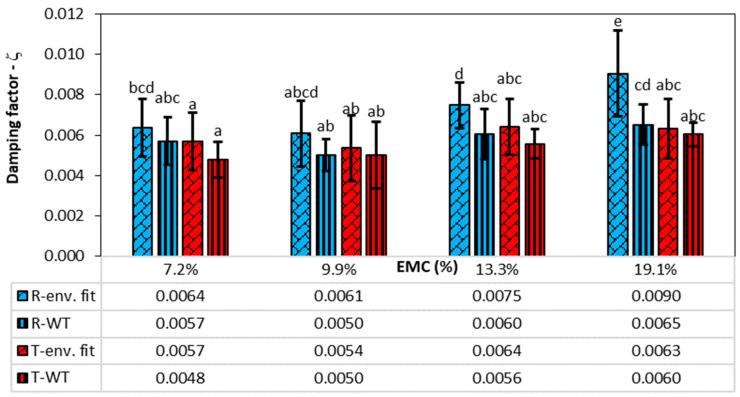
Damping factors at different EMCs, directions of vibration (R—radial, T—tangential), and damping factor determination methods for 1st vibration mode (env. fit—envelope fitting, WT—wavelet transform). The vertical lines represent the standard deviation. The columns with the same letter do not differ significantly from each other at a *p*-value of 0.05.

The damping factors for the first vibration mode obtained from fitting the envelope and the wavelet transform are shown in Figure 3.

The damping factor determined from the time response envelope fit shows the highest values in all sample groups, while the damping determined from the WT shows lower values everywhere, on average 15.9%. The damping in the radial direction is higher than in the tangential direction and generally increases with wood moisture content, except at the lowest wood moisture content of 7.2%, where the damping is slightly higher than at 9.9% moisture content.

For vibrations in the radial direction at an EMC of 7.2%, the damping factor from the envelope and WT is, on average, 0.0064 and 0.0057, respectively, while in the tangential direction, it is 0.0057 and 0.0048, respectively. The damping then decreases slightly at an EMC of 9.9% and then increases with rising moisture content. At the highest EMC of 19.1% in the radial direction, the damping factor from the envelope and WT averages 0.0090 and 0.0065, respectively, and 0.0063 and 0.0060, respectively, in the tangential direction.

Table 1 shows the results of the statistical analysis, which confirms that the method used to determine the damping factor, the vibration direction of the sample, and the EMC significantly influence the damping factors. A post hoc analysis was carried out to determine statistical differences between the groups, which can be seen in Figure 3. Groups that do not differ statistically at a *p*-value of 5% are labelled with the same letters. According to the analysis, the damping factors determined by envelope fitting and WT do not differ significantly in the radial direction for EMCs of 7.2% and 9.9%, while they differ significantly only for EMCs of 13.3% and 19.1%. In the tangential direction, in contrast, the damping factors determined with the two methods do not differ significantly for all EMCs.

Despite the obvious tendency for damping to increase with the moisture content of the wood, the post hoc analysis showed that most groups did not differ significantly from each other when using the same method to determine damping, nor did they differ in the direction of vibration. Thus, there is only a statistically significant difference between the 19.1% EMC and the other EMC for the envelope fitting method in the radial direction of vibration and between the 19.1% EMC and the 9.9% EMC for the WT method in the radial direction.

Irrespective of the differences in the values of the damping factors determined using the various methods, the results are consistent with the literature, which has determined that the damping factor increases with wood moisture content [7,33]. An initial decrease in damping with increasing EMC and then an increase is also reported [5,6], where the damping factor for spruce with 12% EMC was 0.0042.

Similar results were obtained by Buchelt et al. [12], who determined lower EMC values at similar relative humidities. For example, the EMCs at 26%, 50%, 65%, and 85% RH were 3.2%, 7.8%, 11.6%, and 16.9%, respectively, while in our study at 20%, 44%, 76%, and 88% RH, the EMCs were 7.2%, 9.9%, 13.3%, and 19%, respectively. Buchelt, therefore, gives damping as tanδ of 0.00734, 0.00642, 0.00736, and 0.0095 at the above EMCs, corresponding to damping factors of 0.00367, 0.00321, 0.00368, and 0.00475, respectively. The resulting damping is lower than our values, and Buchelt does not specify the direction of vibration, while the dependence of damping on EMC is the same as in our study, i.e., damping first decreases and then increases with EMC.

**Figure 4:**
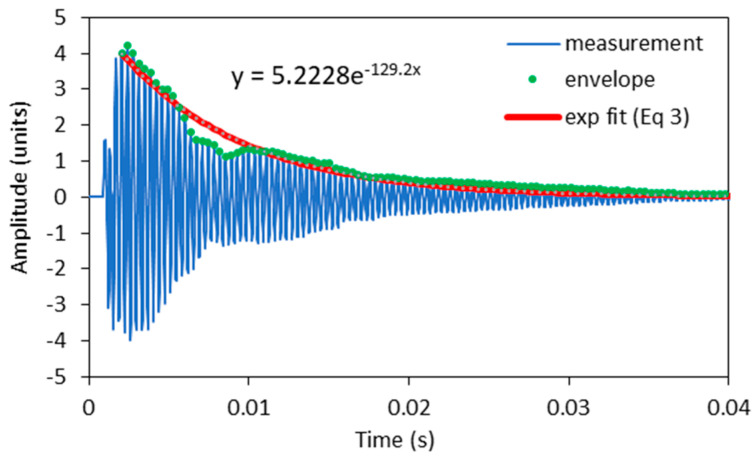
Time response for a sample with 7.2% EMC vibrating in the radial direction.

**Figure 5:**
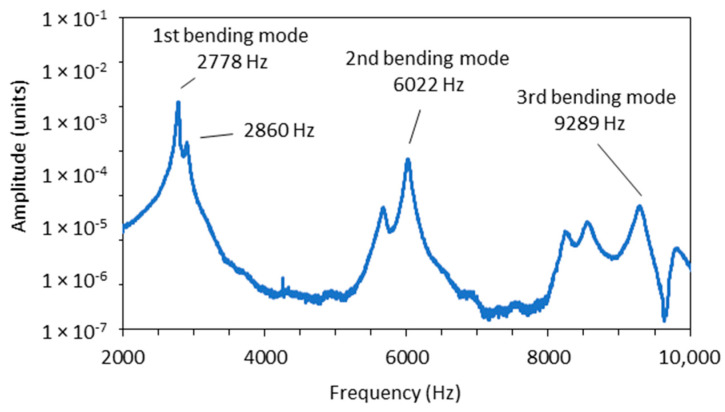
Frequency spectrum of the time response from Figure 4.

To identify the differences between the damping factors determined with the envelope curve of the vibration amplitudes and the WT method, the individual measurements in which there were significant differences in the damping factor were analysed in detail. A typical example of such a case is shown in Figure 4. The figure shows the vibration of a sample with 7.2% EMC vibrating in the radial direction at a natural frequency of 2778 Hz. The latter can be seen in the frequency spectrum in Figure 5, where an additional frequency of 2860 Hz with a significantly lower amplitude is also present and causes the beating of the time response in Figure 4. Further analysis revealed that the frequency of 2860 Hz belongs to the torsional vibrations of the sample. This means that when the sample was excited in the radial direction, a slight torsional vibration was also excited. In several further experiments, attempts were made to excite only the vibration in the radial direction, but a slight torsional vibration was almost always present. Despite the small amplitude of the torsional vibration, it contributes significantly to the error in determining the damping factor, as the amplitudes of the torsional vibration are added to the amplitudes of the bending vibration. Figure 4 also shows the envelope points of the vibration amplitudes together with the exponential fit, which was determined using the least squares method. From this, the damping factor was determined using Equation (3) and was 0.007.

**Figure 6:**
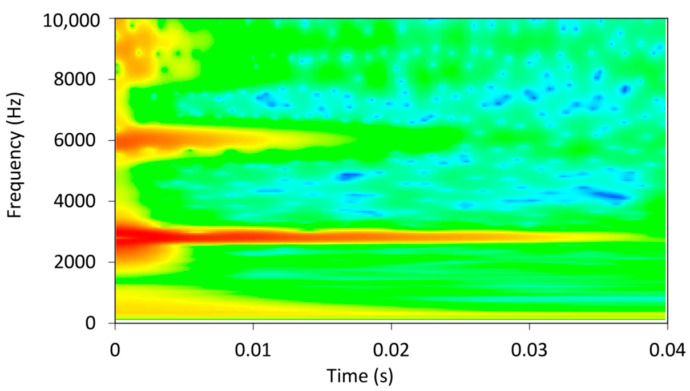
Wavelet transform for time response from Figure 4. The color represents relative amplitude of vibration.

**Figure 7:**
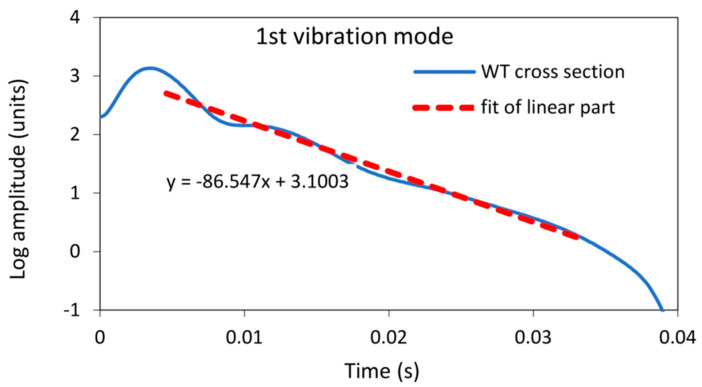
A semi-logarithmic plot of the cross-section of the wavelet transform amplitude from Figure 6, together with the fit of the linear part for a first vibration mode vibrating at a frequency of 2778 Hz.

The damping factor determined by the wavelet transform for the same sample was 0.00496. The contour plot of the WT can be seen in Figure 6, where the decay of the amplitude of the natural vibration at a frequency of 2778 Hz is clearly visible. The cross-section of the wavelet transform for the values of the dilation related to the analysed modal frequencies of 2778 Hz, plotted in a semi-logarithmic plot, is shown in Figure 7. The damping factor determined from the slope of the linear part using Equation (18) is 0.00496, which is significantly less than 0.0074 and determined from the envelope fit of the time response. This is because the WT only takes into account the vibrations at 2778 Hz, while other frequencies have been filtered out.

**Figure 8:**
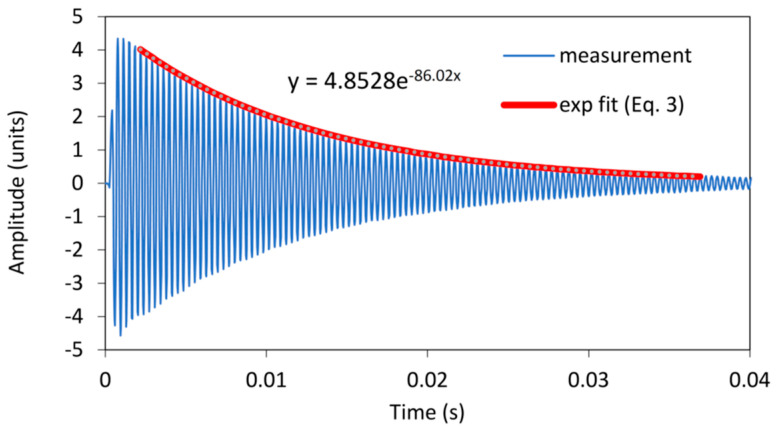
The time response of the sample in which the damping factor determined from the envelope fit was similar to the damping factor determined from the WT.

**Figure 9:**
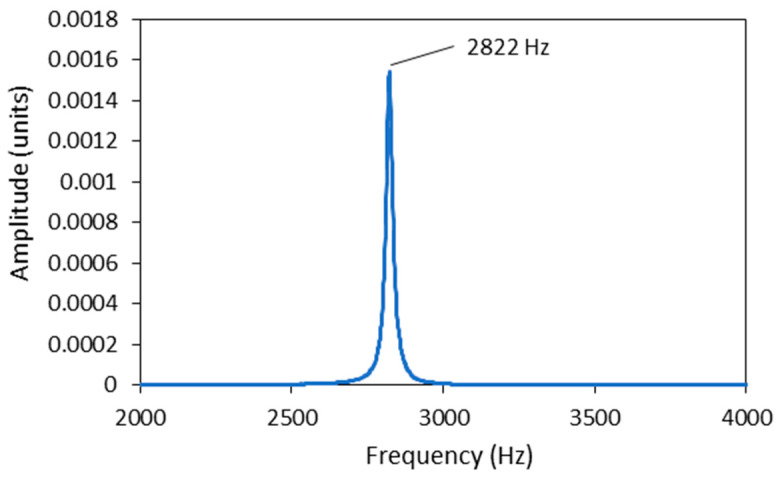
Frequency spectrum of time response from Figure 8.

It should be noted that in some samples, the torsional vibration was not excited, and only the fundamental natural frequency of the first bending vibration mode was present. In this case, the damping factors determined with the envelope fit and the WT method were almost the same. There were very few such cases, as in most instances, a torsional vibration was excited in addition to the bending vibration, which could not be avoided due to the square cross-section of the specimen. A good example of this can be seen in Figure 8 and Figure 9, the first showing the time response with the natural frequency of 2822 Hz together with the fitted envelope, and the second showing the frequency spectrum. The corresponding damping factor determined from the envelope fit and wavelet transform was 0.0048 and 0.0046, respectively.

### 3.2. Damping Factor in Higher Vibration Modes

**Figure 10:**
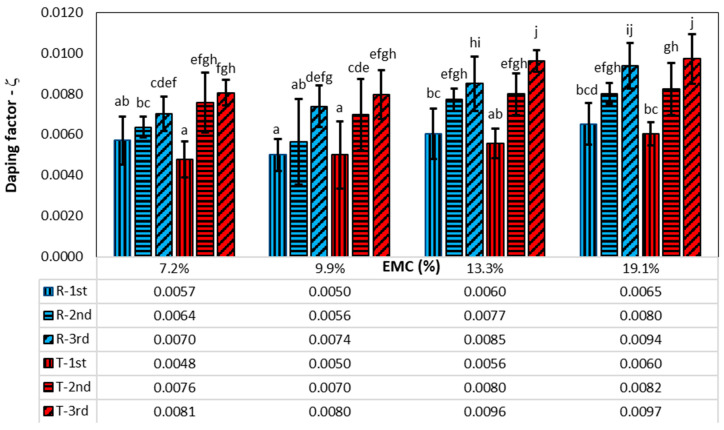
Damping factors at different EMCs, directions of vibration (R—radial, T—tangential), and vibration modes (1st, 2nd, 3rd). The vertical lines represent the standard deviation. The columns with the same letter do not differ significantly from each other at a *p*-value of 0.05.

Figure 10 shows the damping factors of the first three vibration modes, which were determined using the WT method, as the envelope curve for the second and third vibration modes could not be evaluated due to the dominant amplitude of the first vibration mode in the time behaviour. Table 2 shows the results of the statistical analysis. The magnitude of the damping factor is significantly influenced by the vibration mode, the direction of vibration, and the moisture content of the wood.

Figure 10 also shows the results of the post hoc analysis. Groups that do not differ significantly at *p* = 5% are labelled with the same letter. The damping factors have the lowest values for the first vibration mode and then increase for higher vibration modes in both radial and tangential directions. The average values of the damping factors for the first vibration mode in the radial direction are higher than the damping factors in the tangential direction by about 9%, although the statistical analysis shows that there are no significant differences between them for all EMCs. However, the opposite is true for the higher vibration modes: the damping factors in the tangential direction are greater than the damping factors in the radial direction, namely 10.3% for the second vibration mode and 8.8% for the third vibration mode.

The average ratio of the damping factors between the second and first and the third and first vibration modes is 1.2 and 1.42 in the radial direction and 1.49 and 1.72 in the tangential direction. The statistical analysis showed no significant influence of EMC on the ratios of the damping factor, while the vibration direction had a significant influence. There is also a significant difference between the ratios for the third-to-first and the second-to-first modes of vibration.

Mania and Skrodzka [9] came to a similar conclusion when the damping of unmodified and modified spruce wood for the first 10 vibration modes using the 3 dB method was determined. For unmodified spruce wood with a moisture content of 9.5%, Mania determined the 1st, 2nd, and 3rd vibration mode damping factors of 0.0047, 0.0048, and 0.0060, respectively. The values for the first vibration mode are similar to ours, while the damping factors for the second and third vibration modes are much lower than in this study.

The results of this study thus represent an important contribution to the understanding of damping in wood, especially in industries where damping plays an important role. One such industry is certainly the music industry, for which damping is an important factor in the manufacture of musical instruments.

Many studies investigate the damping of different tree species under various wood moisture content and wood modifications. Based on the damping factor, the authors then calculate various coefficients that are used to categorise the tree species into groups. A very popular indicator among researchers is the Acoustic Conversion Efficiency (ACE), which refers to the sound energy emitted by a musical instrument and is calculated from the modulus of elasticity, wood density, and damping factor [4]. Various authors have thus categorised several tree species into groups with different damping or ACE values [4,6,7,15], generally not indicating the direction of vibration, where the damping value of the first vibration mode was taken. For a more precise categorisation, however, the damping of the higher vibration modes should also be taken into account.

## 4. Conclusions

This work used the WT method to determine the damping at different moisture contents of the spruce wood for the first three vibration modes. In addition, the envelope fitting method was used for the first vibration mode. The following conclusions can be drawn from the results:

- The damping factor determined with the wavelet transform is more accurate than the logarithmic decrement or the envelope fitting method when coupling with other modes is present since the wavelet transform allows the decoupling of MDOF systems into single modes and serves as a filter that also filters out the noise added during the measurement.
- The damping factor increases with the EMC for all vibration modes and vibration directions.
- The damping factors for the first vibration mode, determined by fitting the envelope, showed, on average, 15.9% higher values than the damping factors determined with the WT method, which is mainly due to the higher amplitudes resulting from the coupling with other vibration modes.
- The damping factor in the first vibration mode is, on average, 9% higher in the radial direction than in the tangential direction, while the opposite is true for the higher vibration modes. Thus, the damping factor in the tangential direction is higher than in the radial direction by 10% and 8.8% for the second and third vibration modes, respectively.

## Figures and Tables

**Table 1:** Results of statistical analysis for the 1st vibration mode.

	Type III Sum of Squares	df	Mean Square	F	*p*
Determination method	3.75 × 10^−5^	1	3.75 × 10^−5^	21.019	0.000
Vibration direction	2.86 × 10^−5^	1	2.86 × 10^−5^	16.041	0.000
EMC	5.90 × 10^−5^	3	1.97 × 10^−5^	11.006	0.000

**Table 2:** Results of statistical analysis for the first three vibration modes.

Source	Type III Sum of Squares	df	Mean Square	F	*p*
Vibration mode	0.000306	2	0.000153	120.487	0.000
Vibration direction	6.99 × 10^−6^	1	6.99 × 10^−6^	5.510	0.020
EMC	0.000105	3	3.5 × 10^−5^	27.624	0.000

## Data Availability

Additional data is available from the author.

## References

[1] Thomson W.T. (2018). Theory of Vibration with Applications.

[2] Piersol A., Paez T. (2009). Harris’ Shock and Vibration Handbook.

[3] Zhang L., Tiemann A., Zhang T., Gauthier T., Hsu K., Mahamid M., Moniruzzaman P.K., Ozevin D. (2021). Nondestructive assessment of cross-laminated timber using non-contact transverse vibration and ultrasonic testing. Eur. J. Wood Wood Prod..

[4] Brémaud I. (2012). Acoustical properties of wood in string instruments soundboards and tuned idiophones: Biological and cultural diversity. J. Acoust. Soc. Am..

[5] Brémaud I., El Kaïm Y., Guibal D., Minato K., Thibaut B., Gril J. (2012). Characterisation and categorisation of the diversity in viscoelastic vibrational properties between 98 wood types. Ann. For. Sci..

[6] Brémaud I., Gril J. (2021). Moisture content dependence of anisotropic vibrational properties of wood at quasi equilibrium: Analytical review and multi-trajectories experiments. Holzforschung.

[7] Ahmed S.A., Adamopoulos S. (2018). Acoustic properties of modified wood under different humid conditions and their relevance for musical instruments. Appl. Acoust..

[8] Zauer M., Kowalewski A., Sproßmann R., Stonjek H., Wagenführ A. (2016). Thermal modification of European beech at relatively mild temperatures for the use in electric bass guitars. Eur. J. Wood Wood Prod..

[9] Mania P., Skrodzka E. (2020). Modal parameters of resonant spruce wood (*Picea abies* L.) after thermal treatment. J. King Saud Univ.-Sci..

[10] Danihelová A., Vidholdová Z., Gergeľ T., Kružlicová L.S., Pástor M. (2022). Thermal Modification of Spruce and Maple Wood for Special Wood Products. Polymers.

[11] Stanciu M.D., Sova D., Savin A., Ilias N., Gorbacheva G.A. (2020). Physical and mechanical properties of ammonia-treated black locust wood. Polymers.

[12] Buchelt B., Krüger R., Wagenführ A. (2023). The vibrational properties of native and thermally modified wood in dependence on its moisture content. Eur. J. Wood Wood Prod..

[13] Wegst U.G.K. (2006). Wood for sound. Am. J. Bot..

[14] Merhar M., Humar M. (2020). The influence of wood modification on transfer function of a violin bridge. Drv. Ind..

[15] Sproßmann R., Zauer M., Wagenführ A. (2017). Characterization of acoustic and mechanical properties of common tropical woods used in classical guitars. Results Phys..

[16] Gurău L., Timar M.C., Coșereanu C., Cosnita M., Stanciu M.D. (2023). Aging of Wood for Musical Instruments: Analysis of Changes in Color, Surface Morphology, Chemical, and Physical-Acoustical Properties during UV and Thermal Exposure. Polymers.

[17] Mihalcica M., Stanciu M.D., Teodorescu H.D., Iftimie N. (2022). Evaluation of viscous-elastic properties of resonance maple wood. Int. J. Mod. Manuf. Technol..

[18] Jiang X., Wang J., Wang Z., Hua F., He S., Lu B., Wang X., Zhang X., Leng W. (2022). Microstructural and Thermo-Mechanical Characterization of Furfurylated Douglas Fir. Polymers.

[19] Staszewski W.J. (1997). Identification of Damping in Mdof Systems Using Time-Scale Decomposition. J. Sound Vib..

[20] Morlet J. (1983). Sampling theory and wave propagation. Issues in Acoustic Signal/Image Processing and Recognition.

[21] Chui C.K. (1992). An Introduction to Wavelets.

[22] Ruzzene M., Fasana A., Garibaldi L., Piombo B. (1997). Natural frequencies and dampings identification using wavelet transform: Application to real data. Mech. Syst. Signal Process..

[23] Tomac I., Slavič J. (2023). Morlet-wave-based modal identification in the time domain. Mech. Syst. Signal Process..

[24] Chandra N.H., Sekhar A.S. (2016). Nonlinear damping identification in rotors using wavelet transform. Mech. Mach. Theory.

[25] Chen S.-L., Liu J.-J., Lai H.-C. (2009). Wavelet analysis for identification of damping ratios and natural frequencies. J. Sound Vib..

[26] Wang S., Zhao W., Zhang G., Xu H., Du Y. (2021). Identification of structural parameters from free vibration data using Gabor wavelet transform. Mech. Syst. Signal Process..

[27] Perez-Ramirez C.A., Amezquita-Sanchez J.P., Adeli H., Valtierra-Rodriguez M., Camarena-Martinez D., Romero-Troncoso R.J. (2016). New methodology for modal parameters identification of smart civil structures using ambient vibrations and synchrosqueezed wavelet transform. Eng. Appl. Artif. Intell..

[28] Ashory M.R., Khatibi M.M., Jafari M., Malekjafarian A. (2013). Determination of mode shapes using wavelet transform of free vibration data. Arch. Appl. Mech..

[29] Slavič J., Simonovski I., Boltežar M. (2003). Damping identification using a continuous wavelet transform: Application to real data. J. Sound Vib..

[30] Torrence C., Compo G.P. (1998). A Practical Guide to Wavelet Analysis. Bulletin of the American Meteorological Society.

[31] Daubechies I. (1990). The Wavelet Transform, Time-Frequency Localization and Signal Analysis. IEEE Trans. Inf. Theory.

[32] Tchamitchian P., Torresani B. Ridge and skeleton extraction from the wavelet transform. Proceedings of the CBMS-NSF Conference W‘avelets and Their Applications’.

[33] Obataya E. (2017). Effects of natural and artificial ageing on the physical and acoustic properties of wood in musical instruments. J. Cult. Herit..

